# Assessment of Triglyceride Glucose Index in Type 2 Diabetes Mellitus Patients With and Without Cardiac Autonomic Neuropathy

**DOI:** 10.7759/cureus.42541

**Published:** 2023-07-27

**Authors:** Angeline Jeyaseeli, Ganesan R, Dhibika Mathivanan, Allen Prabagaran

**Affiliations:** 1 Physiology, Sri Venkateswaraa Medical College Hospital and Research Centre, Puducherry, IND; 2 Microbiology, Indira Gandhi Medical College and Research Institute, Puducherry, IND; 3 Orthopaedics, Christian Medical College, Vellore, Vellore, IND

**Keywords:** type 2 diabetes mellitus, triglyceride glucose index, fasting blood glucose, dyslipidemia, cardiac autonomic neuropathy

## Abstract

Background

Cardiac autonomic neuropathy (CAN) is a major complication of type 2 diabetes mellitus (T2DM). Hyperglycaemia and hypertriglyceridemia are known risk factors in the development of CAN with T2DM. The triglyceride glucose (TyG) index is calculated using both the fasting blood glucose (FBG) and fasting triglyceride levels (FTG). There is a paucity of literature revealing a direct relationship between the TyG index and CAN in T2DM patients of the south Indian population.

Objective

To assess the TyG index levels in T2DM with and without CAN.

Methods

A cross-sectional study was performed, involving 100 T2DM patients (58 males and 42 females) aged between 30 and 60 years, who attended medicine OPD, Sri Venkateswaraa Medical College, Hospital and Research Centre (SVMCH & RC) during the study period. Age, duration of illness, height, weight, waist circumference (WC), hip circumference (HC), body mass index (BMI), systolic blood pressure (SBP) and diastolic blood pressure (DBP) were measured. Glycated hemoglobin (HbA1C) and lipid profile values were taken from patients' recent medical records. Ewing autonomic function tests were used to diagnose CAN, which included heart rate response to standing, heart rate response to deep breathing, heart rate response to Valsalva maneuver, blood pressure response to standing and blood pressure response to isometric handgrip. FBG and FTG were measured and the TyG index was calculated from these parameters. Statistical Product and Service Solutions (SPSS) (IBM SPSS Statistics for Windows, Version 20.0, Armonk, NY) was used for the statistical analysis and a ‘P’ value < 0.05 was considered statistically significant.

Results

In our study, out of 100 T2DM patients, 42 patients were diagnosed with CAN. The mean levels of TyG Index, HbA1C, FBG, FTG, BMI and WC were significantly (p<0.05) higher in T2DM patients with CAN when compared to T2DM without CAN. We couldn't find any significant difference (p<0.05) in age, duration of illness, blood pressure and lipid profile parameters between the groups.

Conclusion

We found that abdominal obesity, hyperglycemia, and hypertriglyceridemia are the risk factors for developing CAN in T2DM patients. Our study results also showed that the TyG index can be used to predict CAN in T2DM patients.

## Introduction

Type 2 diabetes mellitus (T2DM) is a metabolic disorder characterized by insulin resistance (IR) [1]. India has a diabetic population of 50 million and this will increase to 134 million by 2045 [2]. The complications of T2DM include cardiac autonomic neuropathy (CAN), retinopathy, and nephropathy. [3]. In India, the average annual direct and indirect expenditures for diabetes care were estimated to be Rs. 25,391 and Rs. 4,970 respectively [4]. CAN is a condition which is characterized by cardiac autonomic nerve dysfunction in patients with type T2DM [5]. Initially, CAN often goes undiagnosed since it only becomes symptomatic in the later stage of the disease. Early identification of T2DM patients with CAN is important since it can increase their cardiovascular morbidity and mortality [6,7]. Ewing autonomic function tests are the standard test for diagnosing CAN in T2DM patients. They are simple to perform and have distinctive sensitivity, specificity and reproducibility [8], The modifiable risk factors for CAN are hyperglycemia, obesity and hypertriglyceridemia [9]. The triglyceride glucose (TyG) in­dex is a metabolic index, which is calculated using the product of the fasting glucose and fasting triglyceride levels [10]. It is an accepted marker of IR [1]. TyG index can be used as a marker for predicting T2DM [11] and also can be used as a marker for predicting cardiovascular events in T2DM [12]. The TyG index is independently associated with the development of diabetic nephropathy (DN) among patients with T2DM [13]. However, there is paucity of literature revealing a direct relationship between the TyG index and CAN in T2DM patients for a south Indian population. Therefore, our study aimed to assess the levels of the TyG in­dex in T2DM patients with and without CAN.

## Materials and methods

Study design

The study was conducted in the Department of Physiology, Sri Venkateshwaraa Medical College Hospital and Research Centre (SVMCH&RC). It was a cross-sectional study, which involved 100 T2DM patients (58 males and 42 females) aged between 30 years and 60 years. These patients had attended medicine OPD, SVMCH&RC, and they were willing to bear the cost of testing fasting blood glucose (FBG) and fasting triglyceride (FTG) on the days of recording. We have included patients with a narrow age group between 30 and 60 because patients with more than 60 years would have altered autonomic function tests and it is uncommon to find a patient with T2DM less than 30 years in our demographic area. The sample size was estimated using the assumption that 80.39% of the asymptomatic south Indian T2DM population will have CAN [14]. In this study, CAN was diagnosed by clinical and biochemical investigations other than the TyG index. The sample size was estimated using the formula 4 pq/E², where prevalence (p) = 80.39% q = 19.61%, relative error (E) = 10% of prevalence, and with a confidence interval of 95%.

Selection of subjects

Inclusion Criteria

T2DM patients aged between 30 and 60 years, who attended medicine OPD, SVMCH&RC, and were willing to bear the cost for FBG and FTG tests by themselves on the days of recording.

Exclusion Criteria

Insulin resistance is a major risk factor for developing T2DM [1]. Previous studies have shown that the TyG index is a marker for IR and also showed that it can be used to predict T2DM [1,11] and its complications [12,13]. The risk factors for type 1 diabetes mellitus, gestational and secondary diabetes are multifactorial and hence they were excluded from our study considering them as confounders. Diabetic patients on pacemakers or on anti-arrhythmic drugs, diabetic patients with self-reported neurological or cardiac diseases, diabetic patients with co-morbid illnesses, such as hypertension, hypothyroidism, asthma, chronic obstructive pulmonary disease and chronic renal failure, diabetic patients using drugs, such as beta-blockers, anti-histamines, anti-tussive, anti-depressants, or diuretics and non-complying diabetic patients who did not consent to participate in the study were excluded.

Experimental design

Before the start of the study, ethical clearance was obtained from the Institutional Ethics Committee (human studies) of SVMCH&RC, with registration number: 107/SVMCH/IEC-Cert/Nov 21. The study was conducted in the Physiology research laboratory at SVMCH&RC. Each participant was informed of the procedure in the medicine OPD and asked to provide their informed written consent. On the day of recording, patients were asked to report to the hospital by 8:00 a.m. with overnight fasting. Blood samples for FBG and FTG were taken from them. Then, the patients were asked to report to the Physiology laboratory for autonomic function testing. They were requested to abstain from smoking and drinking coffee/tea, at least 1 hour before the recording of autonomic function testing. Age, duration of diabetes, height, weight, waist circumference (WC), Hip circumference (HC), body mass index (BMI), and waist hip ratio (WHR) were all recorded, with their BMI being calculated using the Quetelet's index of weight (kg) /height (m)^2^ [15]. Then, the patients were provided with 10 minutes of supine rest, to minimize anxiety, following which their stable basal supine blood pressure and heart rate were recorded. Next, Ewing autonomic function tests were performed to confirm the diagnosis of CAN [12], which consists of five autonomic function tests: heart rate responses to deep breathing, heart rate response to stand­ing, heart rate response to the Valsalva maneuver, blood pressure response during posture change, and blood pressure response during Isometric handgrip test. The RR intervals in autonomic function tests were recorded using the Physiopac PP4 (Medicaid, Chandigarh) instrument.

Heart rate responses to deep breathing (E:I ratio)

The patients were asked to perform six cycles of deep, smooth inspiration and expiration, for one minute, while their heart rate was continuously recorded by a lead II electrocardiogram (ECG). The RR ratio interval during the expiration and inspiration (E:I) was measured over the six cycles. The difference between the maximum heart rate and the minimum heart rate was identified as the deep breathing difference (DBD).

A DBD value of more than 15 beats per minute was considered to have a normal parasympathetic function [8].

Heart rate response to stand­ing (30:15 ratio)

The patients were made to remain standing from a supine position while their heart rate was recorded using RR intervals of lead II ECG. The longest RR interval (minimum heart rate) was measured, which normally occurs while standing at around the 30th beat as was the shortest RR interval (maximum heart rate), which occurs around the 15th beat, and the min: max ratio was calculated as the 30:15 ratio.

30:15 ra­tio of < 1.04 was considered to illustrate parasympathetic dysfunction [8].

Heart rate response to the Valsalva maneuver

The patients were asked to forcefully exhale into a sphygmomanometer tube and to maintain a pressure of 40 mmHg in the sphygmomanometer for 15 seconds, while their heart rate was recorded as RR intervals by lead II ECG. The ratio of the longest RR intervals to the shortest RR intervals was calculated and labeled as the Valsalva ratio. A Valsalva ratio above 1.45 was considered to indicate a normal parasympathetic function [8].

Blood pressure response during posture change

The patients were asked to stand up from a supine position and their blood pressure was recorded using a non-invasive blood pressure monitor (OMRON) in both the supine position and immediately after standing. A decrease in the systolic BP of > 20mmHg or a decrease in the diastolic BP of >10 mmHg, while standing, was considered to illustrate an abnormal sympathetic function [8].

Blood pressure response during Isometric handgrip test (ΔDBP mmHg)

The change in BP was measured using a non-invasive blood pressure monitor (OMRON) before and after the subject had performed the hand grip dynamometer maxi­mally for 5 minutes. Here, a rise in diastolic blood pressure (DBP) of <10 mmHg after the release of the handgrip was considered to demonstrate a sympathetic dysfunction [8].

Further, patients will be classified as follows:

1. Non-CAN (Grade 0) - Diabetic patients with normal autonomic function tests

2. Early CAN (Grade 1) - if 1 of 3 parasympathetic tests are abnormal

3. Definite CAN (Grade 2) - if 2 parasympathetic tests are abnormal and

4. Severe CAN (Grade 3) - if 2 parasympathetic are abnormal + 1 or both sympathetic tests are abnormal [16].

Diabetic patients with normal autonomic function tests (Grade 0 mentioned above) were considered as T2DM without CAN and included in Group I, whereas patients with altered autonomic function tests (Grades 1, 2 and 3 mentioned above) were considered as T2DM with CAN and taken in Group II.

Biochemical parameters

A total of 5 ml of venous blood was drawn from the patients to estimate FBG using the Hexokinase method and FTG using the GPO-PAP Enzymatic colorimetric method. Then, the TyG index was calculated using the formula:

TyG index = Ln (fasting TGs (mg/dL)×fasting glucose (mg/dL)/2) [10].

(Ln indicates natural logarithm or logarithm of the base e. Here e is a number which is an irrational number that is approximately equal to 2.72.)

The following parameters glycated hemoglobin (HbA1c) levels, total cholesterol (TC), serum high-density lipoprotein (HDL) and low-density lipoprotein (LDL) were taken from the recent (< three months) medical reports of the study subjects.

Statistical analysis of data

Data were analyzed using Statistical Product and Service Solutions (SPSS) (IBM SPSS Statistics for Windows, Version 20.0, Armonk, NY). The distribution of continuous data such as age, duration of diabetes, height, weight, BMI, WC, HC, WHR, heart rate, systolic and diastolic BP, and TyG index was expressed as mean ± standard deviation. A comparison of the TyG index among T2DM with and without CAN was performed using an independent student’s t-test. A P value < 0.05 was considered statistically significant.

## Results

The mean age, duration of illness, height, weight, BMI, WC, HC, WHR, HR, systolic and diastolic BP, HbA1c levels, serum HDL and LDL, FBG, FTG levels and TyG index were compared between the two groups and shown in Table 1.

**Table 1:** Comparison of baseline parameters in T2DM patients with and without CAN (n=100) Values are expressed as mean (SD); Comparison of variables between groups was conducted by Independent students' t-test. A P value* < 0.05 was considered statistically significant. T2DM: type 2 diabetes mellitus; CAN: cardiac autonomic neuropathy; BMI: body mass index (kg/m^2^); HR: heart rate (bpm); SBP: systolic blood pressure (mmHg); DBP: diastolic blood pressure (mmHg); FBG: fasting blood glucose (mg/dl); FTG: fasting triglyceride (mg/dl); TyG index: triglyceride glucose index; HbA1C: glycated hemoglobin (%); TC: total cholesterol (mg/dl); HDL: high density lipoprotein (mg/dl); LDL: low density lipoprotein (mg/dl)

Variables	T2DM without CAN (n=58)	T2DM with CAN (n=42)	P value*
Age (Years)	50.29±5.36	52.90±4.1	0.117
Duration of illness (Years)	5.83±2.02	6.69±1.93	0.933
Height (cm)	158.02±11.59	157.07±12.40	0.425
Weight (Kg)	69.74±10.56	70.31±10.67	0.859
BMI	27.91±2.8	29.24±7.69	0.000*
Waist circumference (cm)	97.48±3.47	98.60±6.81	0.014*
Hip circumference (cm)	95.86±6.81	93.79±5.87	0.798
Waist Hip Ratio	1.03±0.07	1.04±0.05	0.070
SBP	121.02±9.01	119.94±9.38	0.820
DBP	76.62±8.2	75.12±9.56	0.301
HR	78.06±7.31	77.12±8.27	0.395
HbA1C	7.15±1.23	8.95±1.12	0.05*
TC	191.17±5.19	191.02±4.98	0.591
HDL	44.26±2.97	44.79±2.76	0.507
LDL	146.91±4.45	146.24±5.35	0.075
FBG	164.59±10.96	246.5±24.75	0.000*
FTG	153.28±3.23	246.74±9.11	0.000*
TyG index	9.44±0.04	10.32±0.05	0.002*

## Discussion

In our study, we included 100 patients with T2DM, out of which 58 patients were males and 42 patients were females. Out of 100 patients, 42 patients had altered Ewing autonomic function test results [16]. Subsequently, they were grouped as T2DM patients with CAN. Previous studies performed by Dharmarajan Paneerselvam et al., and Bhuyan AK et al. have shown a high prevalence of CAN in the south and northeast Indian diabetic populations, 80.39% and 70% respectively [14,17]. These variations could be attributed to differences in sample sizes and the characteristics of the populations being studied. CAN increases cardiovascular mortality and morbidity in T2DM patients [7], while the risk factors for the development of CAN in T2DM patients are hyperglycemia, obesity and hypertriglyceridemia [9].

In our study, hyperglycemia was illustrated by the levels of HbA1C and FBG, which were significantly (p<0.05) higher in T2DM patients with CAN, compared to T2DM without CAN. Similarly, the FTG was used as a measure of hypertriglyceridemia and was significantly (p<0.05) higher in T2DM patients with CAN compared to T2DM patients without CAN. BMI and WC which are known indices of obesity were also significantly (p<0.05) higher in T2DM patients with CAN compared to T2DM patients without CAN. Similar observations were made in a study done by Akbar M et al. [18].

IR causes dysregulation in glucose metabolism and leads to T2DM. The TyG index, which was calculated from the FBG and FTG levels, is an accepted marker of IR [1]. A previous study has shown that the TyG index can be used as a marker for predicting T2DM [11]. Thus in patients with T2DM, the TyG index can also be used as a marker for predicting cardiovascular events [12]. The TyG index is independently associated with the development of DN among patients with T2DM [13]. However, the relationship between the TyG index and CAN in T2DM patients has not been extensively studied. Therefore, in our study, we found a significant (p<0.05) difference in the TyG indices between T2DM patients with and without CAN. The TyG index values were higher in T2DM patients with CAN compared to the T2DM patients without CAN. Similar findings were observed in a study performed by Akbar M et al. [18]. The findings in our study suggest that patients with T2DM who are also obese and have relatively poor glycaemic control and high triglyceride levels are at risk of developing CAN. However, in our study, we didn't find any significant difference in TC, LDL and HDL between the groups. Future studies with larger sample sizes done in the general population could reveal the difference in all these parameters between the groups.

Previous studies have also observed that the duration of diabetes, systolic and diastolic BP, and aging-related neuronal death are potential risk factors in T2DM patients for developing CAN [19,20]. In our study, we didn't find any significant difference (p<0.05) between age, duration of diabetes and systolic and diastolic BP for T2DM patients with CAN and without CAN. Conversely, a study performed by Akbar M et al. found a strong association between the above-mentioned parameters and CAN [18]. However, in their study, participants with extremities of age (aged 18 years and above) were included, while hypertensive patients were not excluded from their study. In our study, we have included patients with narrow age groups between 30 and 60, because patients with more than 60 years will have altered autonomic function tests and it is uncommon to find a patient with T2DM less than 30 years in our demographic area. This may be the reason we didn't find any significant difference in age and duration of diabetes between the two groups. We have also not included hypertensive patients with T2DM in our study, since hypertension is an independent risk factor for CAN. So in our study, we didn't find any significant difference in systolic and diastolic BP between the two groups.

Our study has the following limitations; it was a cross-sectional study, which meant that a direct causal relationship could not be established based on the results. Moreover, the patients in our study were recruited from a particular location over a period of time and not from the general population of T2DM. Therefore, prospective cohort studies are needed to assess the TyG index as a marker for predicting CAN in patients with T2DM.

## Conclusions

In our study, we found that abdominal obesity measured by WC and BMI, hyperglycemia measured by HbA1C and FBG, and hypertriglyceridemia measured by FTG are the risk factors for developing CAN in T2DM patients. Our study results also showed that the TyG index can be used to predict CAN in T2DM patients.
